# Generation of G protein-coupled receptor antibodies differentially sensitive to conformational states

**DOI:** 10.1371/journal.pone.0187306

**Published:** 2017-11-01

**Authors:** Andrea S. Heimann, Achla Gupta, Ivone Gomes, Rahman Rayees, Avner Schlessinger, Emer S. Ferro, Ellen M. Unterwald, Lakshmi A. Devi

**Affiliations:** 1 Proteimax Biotechnology LTDA, Av Corifeu de Azevedo Marques, São Paulo, SP, Brazil; 2 Department of Pharmacological Sciences, Icahn School of Medicine at Mount Sinai, New York, United States of America; 3 Department of Pharmacology, Biomedical Science Institute, University of São Paulo, São Paulo, SP, Brazil; 4 Department of Pharmacology, Temple University School of Medicine, Philadelphia, United States of America; Indian Institute of Technology Kanpur, INDIA

## Abstract

The N-terminal region of G protein-coupled receptors can be efficiently targeted for the generation of receptor-selective antibodies. These antibodies are useful for the biochemical characterization of the receptors. In this study, we developed a set of criteria to select the optimal epitope and applied them to generate antibodies to the N-terminal region of 34 different G protein-coupled receptors. The antibody characterization revealed that a subset of antibodies exhibited increased recognition of the receptor following agonist treatment and this increase could be blocked by treatment with the receptor antagonist. An analysis of the epitopes showed that those antibodies that exhibit increased recognition are on average twelve residues long, have an overall net negative charge and are enriched in aspartic and glutamic acids. These antibodies are useful since they facilitate studies examining dose dependent increases in recognition of receptors in heterologous cells as well as in native tissue. Another interesting use of these antibodies is that they facilitate measuring changes in receptor recognition in brain following peripheral drug administration; for example, systemic administration of cocaine, a blocker of dopamine transporter that increases local dopamine levels at the synapse, was found to lead to increases in antibody recognition of dopamine receptors in the brain. Taken together these studies, in addition to describing novel tools to study native receptors, provide a framework for the generation of antibodies to G protein-coupled receptors that can detect ligand-induced conformational changes.

## Introduction

G protein-coupled receptors (GPCRs) play many biological functions by activating distinct and diverse signal transduction pathways in different tissues. These receptors have a common seven transmembrane, heptahelical topology and a well-studied mechanism of signaling primarily involving activation of heterotrimeric G proteins [1]. Compared to the number of studies that examine molecular pharmacological properties, relatively fewer studies have focused on characterizing the cell biological and biochemical properties of GPCRs and the majority of such studies have been with genetically engineered receptors modified with epitope tags [2–4]. The paucity of studies characterizing the biochemical properties of native receptors is largely due to a lack of selective tools to probe specific receptor functions in endogenous tissues. In order to facilitate studies with GPCRs examining the cell biological, biochemical and anatomical properties of native receptors we decided to generate polyclonal antibodies directed against unique epitopes present in the N-terminal region of GPCRs.

Previous studies have suggested that the N-terminal region of GPCRs is suitable for generation of receptor selective antibodies [5–8]. For example, by targeting the N-termini of mu and delta opioid receptors, antibodies were generated that we found to be useful for the characterization of the biochemical and cell biological properties of these receptors [6,7]. Moreover, we found that antibodies against certain epitopes differentially recognized activated (agonist-treated) receptors [6,7]. We also showed that these antibodies recognize the post-activation state of the receptor and this enabled us to probe the spatio-temporal dynamics of receptor activation in the brain following peripheral drug administration [7]. Since antibodies to some (but not all) of the epitopes at the N-terminus of the same receptor exhibited this property, we proposed that the N-terminal region undergoes a conformational change in response to activation that leads to the movement of the glycosylated side chains on residues in the N-terminus resulting in revealing or masking of the epitope [6,7]. We tested this by generating antibodies to the N-terminal region of 6 different family A GPCRs, and found that a portion of the receptor N terminus is masked, and another portion is unmasked upon agonist-induced receptor activation [6]. We also found that the conformation-sensitive antibodies could be used to examine the duration and extent of activation of endogenous receptors [6]. From this we surmised that targeting a specific region in the N-terminus that is proximal to residues that have the potential to be glycosylated (Asn or Ser/Thr) should result in the generation receptor-selective antibodies that recognize conformational changes in native receptors in endogenous tissue. In this study we present data with 38 antibodies to 34 different family A GPCRs that are able to recognize native receptors. When examined for their ability to differentially recognize activated receptors, we found that the majority (but not all) of the antibodies exhibited increased recognition of agonist-treated receptors; none exhibited decreased recognition. Next we analyzed the 38 epitopes in order to predict the optimal epitope sequence that would be differentially recognized by the antibody. Finally, since the highest change in recognition was observed with D2 dopamine receptors we characterized this antibody further. We find that the antibody to D2 dopamine receptors is able to detect the dose-dependent increases in receptor recognition in heterologous cells, in cell lines expressing endogenous receptors and in primary neurons. Furthermore, we find that peripheral administration of cocaine (a dopamine transporter blocker that causes increases in the local concentration of dopamine at the synapse and hence activation of dopamine receptors) leads to a significant increase in the D2 receptor recognition in select brain regions. Taken together these studies show that conformation-sensitive antibodies would also be helpful in the detection of receptors from endogenous tissues and in examining dynamics of receptor activation following drug administration.

## Materials and methods

### Generation of antibodies

38 different antigenic peptides (shown in Table 1) were used to generate antisera against 34 different GPCRs. These peptides were commercially synthesized on a poly-lysine backbone as multiple antigenic peptides (MAPs) [6] by the Tufts University Core Facility. Rabbits were used for the generation of antisera. Prior to immunization, 2 ml of blood was collected from the ear vein of each rabbit for the generation of pre-immune sera. Individual MAPS were dissolved in sterile Tris-buffered saline (TBS) (5 mg/0.5 ml). An emulsion was prepared by placing equal volumes of alumen and MAP solution in an eppendorf tube and vortexing at moderate speed for 20 min. Animals were injected subcutaneously on day 1 with emulsion containing 5 mg MAP, on day 14 with a booster containing 15 mg MAP, on day 28 with a booster containing 25 mg MAP, on day 42 with a booster containing 35 mg MAP. Animals were sacrificed on day 49 by intravenous administration of 0.3 ml pentobarbital through the ear vein. As much blood as possible (~60 ml) was collected from the heart and used to prepare serum as described below.

**Table 1:** Receptor recognition by anti-GPCR antibodies following agonist treatment.

	Receptor Name	Antigen	AA	Agonist	% of control
**1**	**D2 dopamine**	LSWYDDDLER	10	Quinpirole	153.0 ± 6.0
**2**	**Mu opioid**	SDPLAPASWSPAPGSWL	18	DAMGO	151.0 ± 7.0
**3**	**Delta opioid**	LVPSARAELQSS	12	Deltorphin II	150.9 ± 5.0
**4**	**MC4 melanocortin**	NSTHHHGMYTSLHLWN	16	alpha-MSH	145.9 ± 7.0
**5**	**B1 bradykinin**	QAPANITSCE	10	BK 1–8	144.3 ± 6.0
**6**	**Beta2 adrenergic**	SRAPDHDVTQE	11	Isoproterenol	142.0 ± 1.0
**7**	**AT1 angiotensin**	lnsstedgik	10	Angiotensin II	141.0 ± 3.0
**8**	**GPCR55**	PTLSQLDSN	9	AM 251	140.5 ± 9.0
**9**	**Ghrelin (GHSR)**	DLDWDASPGN	10	Ghrelin	140.4 ± 1.0
**10**	**CB1 cannabinoid**	DIQYEDIKGDMA	11	HU210	139.5 ± 5.0
**11**	**5HT**_**1A**_ **serotonin**	TTTSLEPFGTG	11	R(+)-8-OH-DPAT	137.3 ± 2.0
**12**	**Neurotensin**	MEATFLALSL	10	Neurotensin	130.6 ± 6.0
**13**	**Vasopressin V1b**	SEPSWTATPS	10	Vasopressin	129.7 ± 5.0
**14**	**Cholecystokinin 1**	VVDSLLMNGSNI	12	CCK8	127.7 ± 2.0
**15**	**Beta1 adrenergic**	PLPDGAATAARL	12	Isoproterenol	127.6 ± 4.0
**16**	**B2 bradykinin**	NCPDTEWWSWLNA	13	BK	125.4 ± 2.0
**17**	**A2A adenosine**	GTEAPGGGTRATPYS	15	UK14304	125.4 ± 6.0
**18**	**M2 muscarinic**	NNGLAITSPY	10	Bethanecol	125.4 ± 8.0
**19**	**CB2 cannabinoid**	GLEFNPMKEYMI	11	HU210	125.3 ± 6.0
**20**	**NK1 (substance P)**	VLPMDSDLFP	10	Substance P	124.5 ± 2.0
**21**	**Neuropeptide Y1**	TLFSRVENYSVHYNV	15	NPY	124.0 ± 6.0
**22**	**AT2 angiotensin**	DNFSFAATSR	10	Angiotensin II	119.9 ± 4.0
**23**	**Alpha1b adrenergic**	DLDTGHNTSAPAH	13	Phenylephrine	116.9 ± 5.0
**24**	**Kappa opioid**	DQQLEPAHISPA	12	Dynorphin A	116.3 ± 3.0
**25**	**D1 dopamine**	NTSTMDEAGLPAERDF	16	SKF-38393	113.1 ± 4.0
**26**	**Mu opioid**	SDPLAPASWSPA	12	DAMGO	101.0 ± 4.0
**27**	**M1 muscarinic**	LAPGKGPWQV	10	Bethanecol	104.0 ± 6.0
**28**	**CB2 cannabinoid**	CRELELTNGSN	11	HU210	102.0 ± 4.0
**29**	**Endothelin (EDNRB)**	EVMTPPTKT	9	ET-1	100.0 ± 8.0
**30**	**NK1 (substance P)**	SNQFVQPTWQ	10	Substance P	99.0 ± 4.0
**31**	**Leukotriene B4R (BLTR1)**	TAATSSPGGM	10	Leukotriene B_4_	100.0 ± 5.0
**32**	**Leukotriene B4R2 (BLTR2)**	SVCYRPPGNE	10	Leukotriene B_4_	98.0 ± 7.0
**33**	**Prostaglandin D2**	NESYRCQTSTWV	12	Prostaglandin D2	103.0 ± 7.0
**34**	**Prostaglandin E2**	NSFNDSRRVE	10	Prostaglandin E2	103.0 ± 7.0
**35**	**Prostaglandin F2**	SINSSKQPAS	10	Prostaglandin F2	100.0 ± 4.0
**36**	**Vasopressin V1a**	DVRNEELAKL	10	Vasopressin	105.0 ± 8.0
**37**	**Vasopressin V2**	STVSAVPG	8	Vasopressin	101.0 ± 6.0
**38**	**Neurotensin**	LDVNTDIYSKVLVT	10	Neurotensin	99.0 ± 3.0

Membranes (5 μg) from HEK-293 cells expressing individual receptors were treated with 1 μM of receptor agonist and changes in receptor recognition were ascertained by ELISA using anti-receptor antibodies as described in Methods. Values obtained with vehicle treatment were taken as 100%. Data represent Mean ± SE, n = 3–6.

Serum preparation: Blood collected before and after immunization was allowed to clot either for 30–60 min at 37°C or 2 h at room temperature. The clot was separated from the sides of the tube with the help of a Pasteur pipette, kept overnight at 4°C and centrifuged for 10 min at 10,000 x g at 4°C. The supernatant was collected and stored in aliquots at -20°C or at 4°C in the presence of a final concentration of 0.01% sodium azide.

Antisera to all 38 MAPs were affinity purified as described below. Whole cell ELISA with HEK293 cells alone or HEK293 cells expressing the various receptors [9] were carried out to measure receptor recognition by the antibodies.

### Antibody purification

All antisera were affinity purified using immobilized epitope peptide column. Briefly, coupling of the immunization peptide to the resin was achieved by incubating 5 mg of the peptide with 4 mL of CarboxyLink Gel slurry (CarboxyLink Coupling Gel (Immobilized Diaminodipropylamine)–Thermo Scientific), 0.5 mL of the Coupling Buffer (0.1M MES buffer [2-(N-Morpholino) ethansulfonic acid], 0.9% NaCl, pH 4.7) and 60 mg EDC for 3 hours. Affinity purification was carried out as described in CarboxyLink™ Immobilization Kit manufacturer’s manual (Thermo Scientific).

### ELISA

All affinity-purified antibodies were tested by ELISA assay against the peptide epitope used to generate the antibodies as well as tissue membrane preparations as described previously [6]. For studies using membrane preparations from cocaine and saline treated animals levels of endogenous D2 dopamine receptors or CB1 cannabinoid receptors were determined by ELISA as described previously [6].

### Agonist-mediated change in recognition by the antibody

Effect of ligand treatment on receptor recognition by the antibodies was assayed by ELISA using either HEK-293 membranes stably expressing individual receptors or SK-N-SH cells endogenously expressing the receptors being probed. For experiments using membranes, 5 μg/well of membranes were plated onto a 96 well high binding microplate (Greiner Bio-One). The next day, wells were washed with phosphate-buffered saline (PBS) and incubated without or with 1μM of agonist ligands in 50 mM Tris-Cl, pH 7.5, for 30 min at 37°C (in the presence of a protease inhibitor mixture; Sigma). Membranes were quickly rinsed three times (within 5 min) with cold PBS and fixed with 3.7% formaldehyde in 1X PBS for 20 min at room temperature, and then washed 5 times with cold PBS. ELISA was carried out by incubating membranes with 1% BSA and 5% sucrose in PBS for 1 h at 37°C, followed by overnight incubation at 4°C with a 1:500 dilution of primary antibody in 1% BSA in PBS. The wells were then washed three times with PBS (5 min each wash) followed by a 1 h incubation at 37°C with 1:500 dilution (in PBS) of secondary antibody coupled to horseradish peroxidase. The wells were washed three times with PBS (5 min each wash), and color was developed by the addition of the substrate, o-phenylenediamine (5 mg/10 ml in 0.15 M citrate buffer, pH 5, containing 15 μl of H_2_O_2_). Absorbance at 490 nm was measured with a Bio-Rad ELISA reader and values obtained in the absence of ligands were taken as 100%.

For experiments using cells, ELISA was carried out as described previously [6,10]. Briefly, SK-N-SH cells (2 x 10^5^) were seeded into each well of a 24-well polylysine coated plate. Next day cells were treated with different concentrations of receptor agonists in 50 mM Tris-Cl, pH 7.5 for 30 min at 37°C. Cells were quickly rinsed 3 times with ice-cold PBS (to remove the agonist) and fixed with ice-cold methanol for 3 min. ELISA was carried out as described above using 1:1000 dilution of primary antibodies and 1:1000 dilution of secondary antibody coupled to horseradish peroxidase. For experiments examining the effect of antagonist treatment cells were pre-treated with 1 μM antagonist for 10 min at 37°C followed by treatment with 100 nM agonist.

### Western blotting

Western blotting analyses were carried out using either whole tissue lysates or membranes preparations as described [6]. Membranes were prepared from rat tissues (heart, kidney and brain) as described previously [11]. Primary cortical neurons were generated as described previously [12] and membranes prepared as described in [11]. Membranes from a given tissue/cell preparation (10–50 μg) were subjected to SDS-PAGE and transferred to nitrocellulose membranes. Nitrocellulose membranes were cut into strips; individual strips were subjected to Western blot analysis by blocking with 1% albumin in PBS and using a 1:500–1:2000 dilution of a primary antibody (1% albumin in PBS) to a GPCR and a 1:15,000 dilution in PBSt (PBS containing 0.1% Tween20) of IR Dye 700 anti-rabbit IgG (Li-Cor Biosciences, Lincoln, NE). Blots were visualized using the Odyssey Imaging system (Li-Cor Biosciences, Lincoln, NE).

### Analysis of the antigenic epitopes

**Fig 1:**
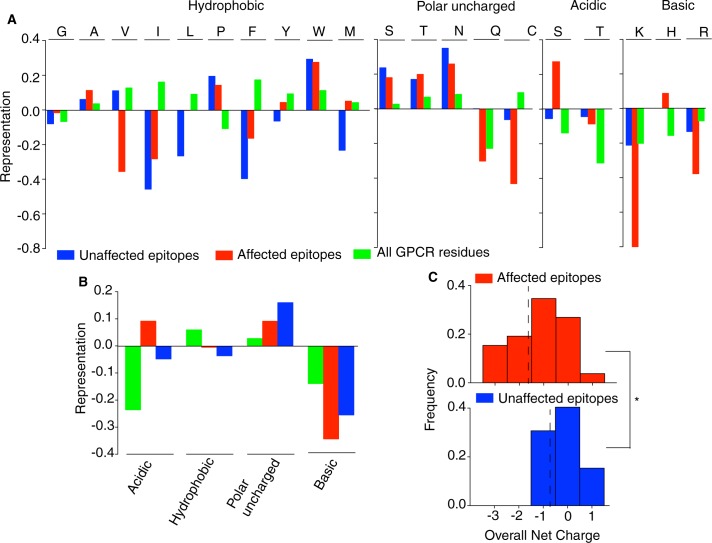
Biophysical features of antigenic epitopes. (**a**) Representation of amino acids in affected epitopes (epitopes with an absorbance greater than 100%) and not affected epitopes (epitopes with an absorbance equal to 100%) and their abundance in GPCRs (**b**) Abundance of negatively, positively, polar and nonpolar residues in affected, not affected epitopes and the GPCR, in general. (**c**) Frequency plot of the overall net-charge of affected and unaffected epitopes. Comparison of the distribution of net charges between unaffected and affected epitopes was carried out by a Wilcox-Man-Whitney rank sum test. *p<0.001.

The amino acid representation of unaffected epitopes (epitopes without any antibody recognition or 100% recognition), affected epitopes (epitopes with greater than 100% recognition), the GPCR and exposed residues on each GPCR was calculated by comparing the amino acid frequencies of the epitopes tested to a representative set of proteins from the human proteome. The solvent accessible residues (‘exposed residues’) on the GPCRs were predicted using the PredictProtein package [13]. The representative set of human proteins was obtained by using the UniqueProt software [14] on the human proteome obtained from UniProt. Using the frequencies of each amino acid in the human proteome, we calculated the log base10 fold change by dividing the frequency of a specific amino acid in an epitope to the frequency of a specific amino acid in the representative set and taking the log base 10 of the value [15] (Fig 1 & S1 Fig). To compute the representation of charged residues in the epitopes we tabulated the frequency of negatively, positively, polar and nonpolar residues in the epitopes and compared them to the corresponding frequencies seen in the representative human proteome protein set (Fig 1B and 1C). We performed a Wilcox-Man-Whitney rank sum test to compare both the distribution of net charges between unaffected epitopes and affected epitopes as well as the lengths of the affected and unaffected epitopes.

### Cell culture and transfection

CHO cells stably expressing Flag-tagged mouse D2 dopamine receptors were grown in F12 media containing 10% FBS, 1% penicillin-streptomycin and 400 μg/ml G418. HEK-293 and SKNSH cells were grown in Dulbecco's modified Eagle's Medium (DMEM) containing 10% FBS and 1% penicillin-streptomycin.

### Animals, drug treatment and tissue preparation

#### Mice

Adult male C57Bl/6 mice (Charles River, Inc. 22–24 g at the start of the experiment) were group housed 4–5 per cage with ad libitum access to water and standard mouse chow. Animals were housed under constant temperature and humidity with a 12h light/dark cycle. The animal facility is AAALAC approved and care of animals is provided by a team of veterinarians and trained technicians. Animal use procedures were conducted in strict accordance with the NIH Guide for the Care and Use of Laboratory Animals and approved by the Institutional Animal Care and Use Committee of Temple University.

Cocaine hydrochloride, generously provided by the NIDA drug supply program was dissolved in 0.9% saline and administered intraperitoneally (i.p. 20 mg/kg body weight). This treatment does not cause suffering or distress to the animals. Mice were sacrificed 30 min later by exposing them to CO_2_ from compressed gas followed by rapid decapitation. Brains were collected, dissected to various regions and membranes prepared as described previously [16].

#### Rabbits

Male California rabbits (6 months old) were individually housed with ad libitum access to water and standard rabbit chow. The rabbits were housed under constant temperature and humidity with a 12-hour light/dark cycle. The animal facility is AAALAC approved and care is provided by a team of veterinarians and trained technicians. Rabbits were used to generate polyclonal antibodies to different GPCRs as described above. These procedures do not cause suffering or distress to the animals. Rabbits were sacrificed by intravenous administration of 0.3 ml of pentobarbital through the ear vein and as much blood as possible (~60 ml) was collected via cardiac drive.

#### Rats

Male Wistar rats (6 months old) were group housed 2 per cage with ad libitum access to water and standard rat chow. Animals were housed under constant temperature and humidity with a 12h light/dark cycle. The animal facility is AAALAC approved and care is provided by a team of veterinarians and trained technicians. Rats were anaesthetized (10 mg xylazine/5mg ketamine/100 g i.p) and perfused through the ascending aorta with 50 ml of 0.9% NaCl containing 100 U/ml of heparin, followed by 60 ml of a mixture of 3.75% of acrolein and 4% paraformaldehyde (PFA) in 0.1 M phosphate buffer (PB), pH 7.4 followed by 40 ml of 4% PFA in PB. The brain was then dissected and post-fixed by immersion in 4% PFA in PB for 1 hour at 4°C. Coronal sections (30 μM thick) were cut on a vibratome in ice-cold PB.

### Immunohistochemistry

For peroxidase staining: Rat coronal sections in PB were stained as described previously [17]. Briefly, sections were treated with 1% sodium borohydride in PB for 30 min to block free aldehyde groups. After several washes in PB the sections were incubated in 0.3% H_2_O_2_ in PB to block endogenous peroxidase activity. Sections were washed several times in PB and blocked for 30 min with 6% bovine serum albumin (BSA) and 5% normal goat serum (NGS) in 0.1 M Tris-buffered saline, pH 7.4 (TBS). Sections were incubated overnight at 4°C with 1:1000 or 1:2000 dilution of anti-receptor antibody in TBS containing 3% NGS and 0.3% Triton-X-100. Control sections were incubated with receptor antisera pre-adsorbed with the corresponding immunogenic MAP (1 h prior to use the anti-receptor antibody was incubated with 1 or 5 mg of the corresponding MAP in PBS containing 0.3% Triton-X-100, 3% NGS and a protease inhibitor cocktail). Following incubation with the primary antibody, sections were washed in TBS and incubated for 2 h at RT with biotinylated goat anti-rabbit IgG diluted 1:100 in TBS containing 1% BSA and then for 1 h with streptavidin-biotin peroxidase. After further washes in TBS, sections were incubated with biotinyated tyramine for 10 min and re-incubated in ABC. Sections were then treated with 0.05% diaminobenzidine in the presence of 0.008% nickel chloride and 0.03% H_2_O_2_ in TBS, washed, mounted on gelatin-coated glass slides, dehydrated in graded ethanols, defatted in xylene, cover slipped with Permount and examined using a Leitz Aristoplan microscope.

For fluorescence staining: Rat coronal sections in PB were blocked for 30 min with 6% bovine serum albumin (BSA) and 5% normal goat serum (NGS) in 0.1 M Tris-buffered saline, pH 7.4 (TBS). Sections were incubated overnight at 4°C with 1:1000 or 1:2000 dilution of anti-receptor antibody in TBS containing 3% NGS and 0.3% Triton-X-100. Sections were washed in TBS and incubated for 2 h at RT with 1:1000 dilution of Alexa 488 conjugated secondary antibody in TBS containing 3% NGS and 0.3% Triton-X-100. Sections were washed in TBS, treated with propidium iodide to stain cell nuclei, mounted on glass slides, cover slipped with Permount and examined using a Leitz fluorescence microscope.

### Statistical analysis

Data was analyzed using t-test or One-Way Anova as appropriate using GraphPad Prism 6.0 software. P <0.05 was considered to be significant.

## Results and discussion

### Selection of epitopes and generation of the antibodies

Since studies with GPCRs have been severely hampered by a lack of antibodies that selectively recognize native receptors in endogenous tissue, we focused on the N-terminal regions of family A GPCRs and generated antibodies to 34 individual receptors. The criteria applied for the selection of the antigenic epitopes were: (i) location within the N-terminal region, (ii) between 9–17 amino acids in length, (iii) most conserved across species, (iv) relatively hydrophilic in nature, (v) next to (but not containing) glycosylatable residues, (vi) contain or be near one or more proline residues, (vii) not containing cysteine (and if present replaced by serine). The epitopes were chosen with the help of the ExPASy software so that it was possible to predict peptide localization with reference to *N*-glycosylation and phosphorylation sites. The peptides were subjected to a Blast search of the NCBI database to ensure that they represent unique sequences and if possible represent a conserved epitope between species. Antigenic peptides corresponding to residues in rat (conserved in human) receptors were commercially synthesized on a poly-lysine backbone so as to generate multiple antigenic peptides and injected into rabbit. The antibodies were purified using an epitope peptide- affinity column and tested by ELISA and Western blotting (and a few were further characterized by immunohistochemistry). We were able to successfully generate antibodies to all 34 individual receptors although in some cases the antibody titers were substantially lower than expected (S1 Table). In a few cases when the first attempt to generate conformational sensitive antibodies failed, we changed the composition of the epitope by either generating a longer epitope (by residue extension) or selecting a different epitope in the N-terminal region; this led to the successful generation of these antibodies (Table 1). Thus conformational sensitive antibodies were obtained following immunization with the SDPLAPASWSPAPGSWL peptide but not with the SDPLAPASWSPA peptide for mu opioid receptor, GLEFNPMKEYMI instead of CRELELTNGSN for cannabinoid 2 receptors, MEATFLALSL instead of LDVNTDIYSKVLVT for neurotensin receptors, and VLPMDSDLFP instead of SNQFVQPTWQ for neurokinin 1 receptors (Table 1).

The antibodies were purified on an epitope- peptide affinity column and the purified antibodies were characterized as to their ability to recognize the receptor in heterologous cells expressing the receptors; control cells not expressing the receptors were used as negative controls. We find that all of our antibodies recognized the cognate receptors as examined by ELISA assays and only in HEK-293 cells expressing the receptor (S1 Table). Next we examined if the antibodies recognized native receptors in endogenous tissues. Western blotting analysis with anti-GPCR antibodies revealed a signal in endogenous tissue (S2 Fig); no signal was detected when antibodies were pre-incubated with the immunizing peptide. We further characterized a subset of these antibodies by immunohistochemical analysis and found that these antibodies can detect a signal only in a brain region or tissue expressing the receptors; select examples are shown in S3 and S4 Figs. Together these results support the idea that a region within the N-terminal tail of GPCRs is suitable for the generation of receptor specific polyclonal antibodies.

### Agonist-treatment mediated enhanced recognition

In a previous study we found that the N-terminal region of opioid receptors undergoes a conformational change following agonist treatment, and this leads to increased or decreased receptor recognition by the antibodies [6,7]. In order to see if the antibodies generated in the present study are able to differentially recognize agonist-treated receptors, we subjected membranes from HEK-293 cells expressing individual receptors to agonist treatment and measured the change in recognition of the receptor by the antibody using ELISA. We find that in most cases the antibodies exhibited increased recognition of agonist treated receptors (Table 1). Thus 19 out of the 38 antibodies exhibited >25% increase in receptor recognition while 13 antibodies did not exhibit measurable changes in receptor recognition (Table 1). Interestingly, the dopamine D2 receptor antibody showed the highest increase in recognition of more than 50% whereas the dopamine D1 receptor antibody showed only a marginal increase of ~ 13% (Table 1). Next, we further characterized the set of antibodies with highest increase in receptor recognition using SK-N-SH human neuroblastoma cells endogeneously expressing the receptors. Agonist treatment led to a dose dependent increase in receptor recognition (EC_50_ in the nM range) and this increase in recognition could be blocked by receptor selective antagonists (Table 2, S5 Fig). It is possible that these antibodies stabilize the activated conformation of the receptor or function as positive allosteric modulators. It should be mentioned that in our studies incubation with the antibodies was carried out under the conditions of minimal signal transduction (ice-cold buffer); further studies directly evaluating signaling by these antibodies would help clarify whether they function as positive allosteric modulators.

**Table 2:** Endogenous receptor recognition by anti-GPCR antibodies following agonist treatment.

Receptor	Agonist	EC_50_ (nM)	E_max_ (% control)	Agonist + antagonist	EC_50_ (nM)	E_max_ (% control)
D2 dopamine	Quinpirole	7.6 ± 1.1	237 ± 8	Quinpirole + Sulpiride	n.a.	121 ± 3
5HT_1A_ serotonin	R(+)-8-OH-DPAT	1.4 ± 2.1	221 ± 8	R(+)-8-OH-DPAT + WAY100135	n.a.	116 ± 16
Cholecystokinin 1	CCK8	0.1 ± 0.4	199 ± 17	CCK8 + SR27987	n.a.	129 ± 24
Ghrelin (GHSR)	Ghrelin	1.8 ± 1.7	184 ± 5	Ghrelin + YIL	n.a.	84 ± 9
Delta opioid*	Deltorphin II	0.6 ± 0.2	182 ± 2	Deltorphin II + TIPPψ	n.a.	103 ± 3
Mu opioid	DAMGO	1.1 ± 0.3	174 ± 9	DAMGO + CTOP	n.a.	98 ± 7
CB1 cannabinoid*	Hu210	4.6 ± 1.3	173 ± 7	Hu210 + SR141716A	n.a.	114 ± 8
Beta2 adrenergic*	Isoproterenol	0.9 ± 1.0	172 ± 5	Isoproterenol + CGP12177	n.a.	100 ± 1
B2 bradykinin	Bradykinin	5.8 ± 1.9	171 ± 7	Bradykinin + WIN64338	n.a.	89 ± 3
AT1 angiotensin*	Angiotensin II	3.9 ± 1.2	170 ± 10	Angiotensin II + losartan	n.a.	108 ± 10
NK1 (substance P)	Substance P	0.8 ± 2.0	146 ± 6	Substance P + L733060	n.a.	55 ± 6

SK-N-SH cells were treated with different concentrations (0–10 μM) of receptor agonist in the absence or presence of antagonist and changes in receptor recognition were measured by ELISA using anti-receptor antibodies as described in Methods. Values obtained with vehicle treatment were taken as 100%. Data represent Mean ± SE, n = 3.

*Values from data in [6]; n.a., not applicable.

### Analysis of the antigenic epitopes

Next, we examined whether changes in recognition by the antibody correlated with the structural properties of epitopes such as residue type, overall net-charge as well as length of the epitope (Fig 1 and S1 Fig). For this we calculated the amino acid representation of unaffected epitopes (epitopes without increased antibody recognition or 100% recognition), affected epitopes (epitopes with greater than 100% recognition), and exposed residues on each GPCR by comparing the amino acid frequencies of the epitopes tested to a representative set of proteins from the human proteome. We found that, in general, residues within the epitope had a distinctive high representation of Alanine and Tryptophan compared to the residues in a representative set of proteins in the human proteome. They also had a distinctive under representation of positively charged residues (such Lysine and Arginine) compared to solvent-exposed residues on the GPCR. Those epitopes that showed increased recognition were further distinguished by the striking over representation of aspartic acid and by a minor overrepresentation of tyrosine, methionine and histidine residues. Additionally, we detected underrepresentation of glutamine and valine in these epitopes as compared to unaffected epitopes. Plotting the representation of negative, positive, polar and nonpolar (at physiological pH) residues against a representative set of proteins from the human proteome reiterated that affected epitopes (i.e. with increased recognition) contain more negatively charged residues than unaffected epitopes (Fig 1B). Due to the high abundance of negatively charged residues in the epitopes for antibodies with increased recognition, we plotted the distribution of overall net charge of both affected and unaffected epitopes (Fig 1C). The epitopes with increased recognition carried a significantly more negative overall net charge compared to the unaffected epitopes (p-value <0.001). Finally, the average length of epitopes with increased recognition was seen to be longer, on average, than unaffected epitopes by two residues (p-value = 0.011). Taken together, we find that affected epitopes (i.e. agonist-mediated increased recognition of a GPCR) are on average, twelve residues in length, have an overall net negative charge, and are enriched in residues such as aspartic acid and glutamic acid. These features could potentially be used to identify epitopes in the N-terminal region of other GPCRs in order to generate antibodies that are differentially sensitive to agonist-activated conformational states of receptors.

### Use of antibodies to detect activity states of endogenous receptors

**Fig 2:**
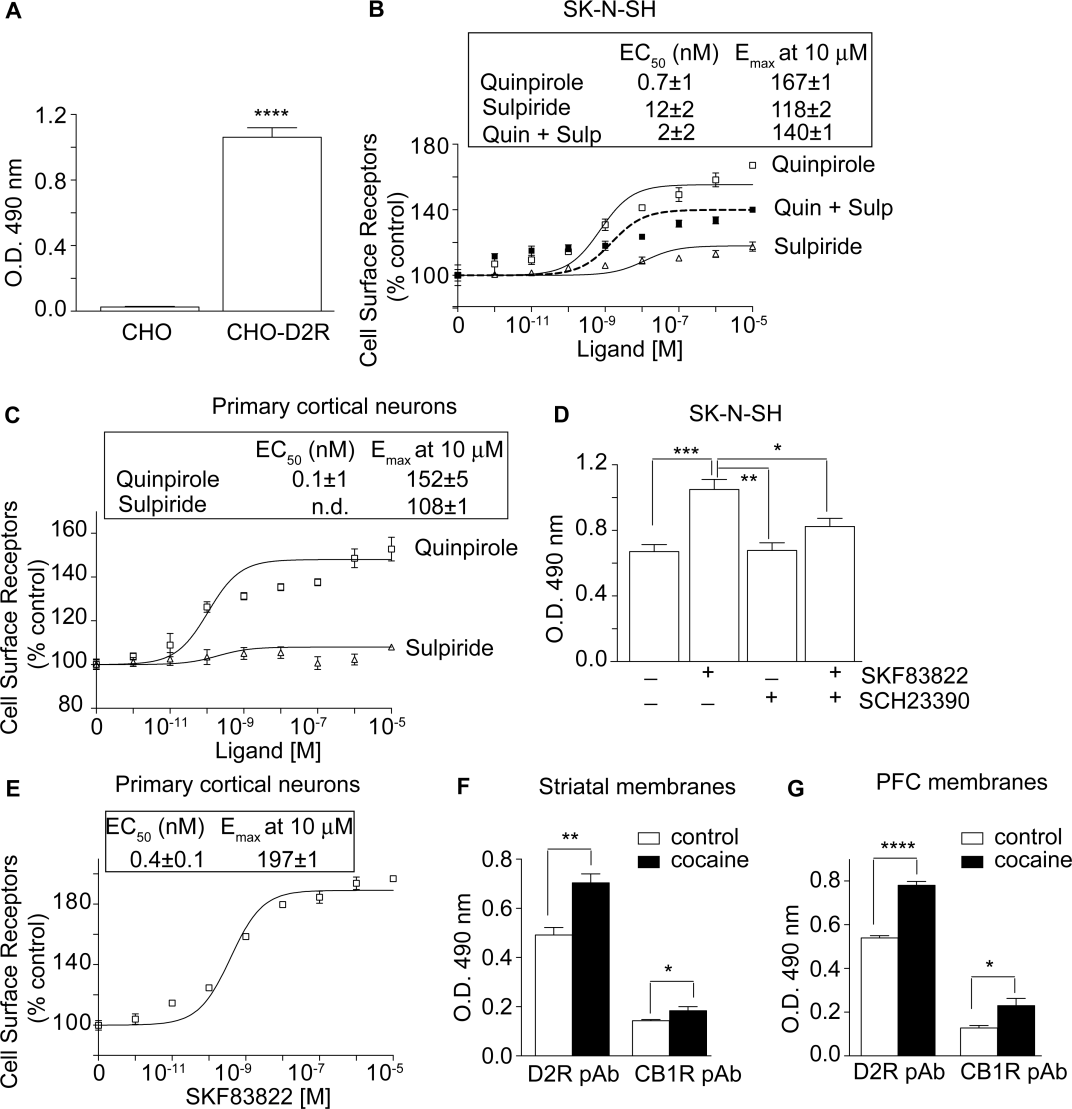
Characterization of polyclonal antibodies to dopamine receptors. **(A)** ELISA with D2R polyclonal antibody detects a signal only in CHO cells stably expressing D2R (CHO-D2R) and not in control CHO cells. Data represent Mean ± SE (*n* = 3). ****p < 0.0001, Student’s t-test. **(B)** SK-N-SH cells or **(C)** primary cortical neurons were treated with various ligands (0–10 μM) and receptor recognition by anti-D2R antibodies measured by ELISA as described under “Experimental Procedures”. **(D)** SK-N-SH cells or **(E)** primary cortical neurons were treated with various ligands (1 μM for panel **D** and 0–10 μM for panel **E**) and receptor recognition by anti-D1R antibodies measured by ELISA. **(F)** Receptor recognition in striatal or **(G)** prefrontal cortex (PFC) membranes from mice treated with vehicle or cocaine (20 mg/ kg body weight) was probed using anti-D2R or anti-CB1R antibodies by ELISA. Data from untreated “controls” were taken as 100%. Statistically significant differences from control are indicated. *p < 0.05, **p < 0.01, ****p < 0.0001; n = 3.

Given that the antibodies described in this study recognize endogenous receptors we next examined whether they could detect receptor conformational changes in endogenous systems. For this we focused on the dopamine receptors and using SKNSH cells endogenously expressing these receptors or primary cortical neurons we examined the effect of ligand treatment on receptor recognition by the antibodies. We find that treatment with the dopamine D2 receptor (D2R) agonist, quinpirole, but not the antagonist, sulpiride, caused a dose-dependent increase in recognition by the anti-D2R antibodies (Fig 2B & 2C). Similarly, treatment with the dopamine D1 receptor (D1R) agonist, SKF83822, but not the antagonist, SHC23390, led to an increase in receptor recognition by anti-D1R antibodies (Fig 2D & 2E). Moreover, we find that antagonist treatment blocked the agonist-mediated increase of receptor recognition (Fig 2). These results indicate that our antibodies can detect changes in native receptor conformation following ligand treatment.

Next we examined if the anti-D2R antibodies could be used to explore the dynamics of receptor activation *in vivo* following administration of drugs of abuse. For this we examined D2 receptor activation in the brain 30 min after peripheral cocaine administration. Cocaine functions by blocking dopamine transporters thereby enhancing synaptic dopamine levels which then leads to activation of dopamine receptors [18]. We find that 30 min following cocaine administration, there is a significant increase in antibody recognition of the D2 receptors in the striatum and prefrontal cortex and the extent of this increase is much greater than that observed for CB1R (Fig 2). These results showing that the D2 receptor activation in the brain can be measured 30 min following peripheral cocaine administration are consistent with data from previous studies that have suggested that the conformation-sensitive antibodies of GPCRs can be used to monitor spatio-temporal dynamics of receptor activity targeting drugs of abuse [6,7].

Taken together, our studies show that the criteria that we used for the identification of antigenic epitopes at the N-terminus of GPCRs can be successfully used to generate antibodies that differentially recognize agonist treated receptors. Moreover, these antibodies can be used to detect the presence of activated receptors *in vivo* following peripheral agonist administration. Thus these antibodies could potentially be developed as biosensors for the activity state of a receptor not only in specific brain regions following drug administration but also in different intracellular compartments. The latter type of studies is supported by reports that used nanobodies to show that treatment with an agonist led to the activation of β2-adrenergic receptors not only at the plasma membrane but also in the endosomal compartment [19].

## Supporting information

S1 Fig Biophysical features of antigenic epitopes. Representation of amino acids in affected epitopes (epitopes with an absorbance greater than 100%), not affected epitopes (epitopes with an absorbance equal to 100%), and residues predicted to be solvent-exposed on the GPCRs tested.
(EPS)

S2 Fig Western blot analysis showing that anti-GPCR antibodies recognize native receptors. Western blots with **(A)** rat brain membranes and anti- adenosine A2A (ADORA 2; epitope: GTEAPGGGTRATPYS) receptor antibodies; **(B)** rat kidney or brain PVN membranes and either anti-angiotensin 1 (AGTR1; epitope: lnsstedgik) or anti-angiotensin 2 (AGTR2; epitope: DNFSFAATSR) receptor antibodies; **(C)** rat brain or heart membranes and either anti-α1B (ADRA1B; epitope: DLDTGHNTSAPAH), anti-β1 (ADRB1;epitope: PLPDGAATAARL) or anti-β2 (ADRB2; epitope: SRAPDHDVTQE) adrenergic receptor antibodies; **(D)** rat heart or brain membranes and either anti-bradykinin 1 (BKR1; epitope: QAPANITSCE) or anti-bradykinin 2 (BKR2; epitope: NCPDTEWWSWLNA) receptor antibodies; **(E)** rat brain membranes and either anti-cannabinoid 1 (CB1R; epitope: DIQYEDIKGDMA), anti-cannabinoid 2 (CBR2; epitope: GLEFNPMKEYM) receptor or anti-GPR55 (epitope: PTLSQLDSN) antibodies; **(F)** rat brain membranes and anti-cholecystokinin (CCK1R; epitope: VVDSLLMNGSNI) receptor antibodies; **(G)** rat brain or heart membranes and either anti-dopamine D1 (DRD1; epitope: NTSTMDEAGLPAERDF) or anti-dopamine D2 (DRD2; epitope: LSWYDDDLER) receptor antibodies; **(H)** rat heart membranes and anti-endothelin B (ET_B_; epitope: EVMTPPTKT) receptor antibodies; **(I)** rat heart membranes and anti-ghrelin (GHSR; epitope: DLDWDASPGN) receptor antibodies; **(J)** rat heart membranes and either anti-leukotriene B4 receptor 1 (BLTR1; epitope: TAATSSPGGM) or anti-leukotriene B4 receptor 2 (BLTR2; epitope: SVCYRPPGNE) antibodies; **(K)** rat brain membranes and anti-melanocortin 4 (MC4R; epitope: NSTHHHGMYTSLHLWN) receptor antibodies; **(L)** rat brain or heart membranes and either anti-muscarinic M1 (M1R; epitope: LAPGKGPWQV) or anti-muscarinic M2 (M2R; epitope: NNGLAITSPY) receptor antibodies; **(M)** rat brain membranes and anti-neuropeptide Y 1 (NPY1R; epitope: TLFSRVENYSVHYNV) receptor antibodies; **(N)** rat kidney membranes and anti-neurotensin (NTSR; epitope: MEATFLALSL) receptor antibodies; **(O)** rat brain membranes and anti-neurokinin 1 (NKR1; epitope: VLPMDSDLFP) receptor antibodies; **(P)** rat brain membranes and either anti-δ opioid (OPRD; epitope: LVPSARAELQSS), anti-κ opioid (OPRK; epitope: DQQLEPAHISPA) or anti-μ opioid (OPRM; epitope SDPLAPASWSPAPGSWL) receptor antibodies; **(Q)** rat brain or kidney membranes and either anti-prostaglandin D2 (PTGDR; epitope: NESYRCQTSTWV), anti-prostaglandin E2 (PTGER; epitope: NSFNDSRRVE) or anti-prostaglandin F2 (PTGFR; epitope SINSSKQPAS) receptor antibodies; **(R)** rat brain membranes and anti-serotonin 5HT1A (5HTR1A; epitope: TTTSLEPFGTG) receptor antibodies; **(S)** rat kidney or heart membranes and either anti-vasopressin V1a (AVPR1A; epitope: DVRNEELAKL), anti-vasopressin V1b (AVPR1B; epitope: SEPSWTATPS) or anti-vasopressin V2 (AVPR2; epitope: STVSAVPG) receptor antibodies. Representative blots of 3 experiments shown. Lane 1: molecular weight markers; Lane 2; 20–50 μg membrane protein.
(EPS)

S3 Fig Immunohistochemical evaluation of anti-GPCR antibodies without or with pre-adsorption with corresponding MAP. Coronal sections from rat cerebral cortex (**A**-**D**), rat cerebellum (**E, F**) or rat periaqueductal gray (**G, H**) were incubated with either anti-DRD2 (A, B), anti-CB1R (C, D), anti-OPRD (E, F) or anti-OPRM (**G, H**) antibodies with or without pre-adsorption with the corresponding MAP (5mg/ ml for B, D, F, and 1 mg/ml for H) followed by incubation with HRP-labeled secondary antibodies as described in Methods.
(EPS)

S4 Fig Immunohistochemical evaluation of anti-GPCR antibodies. Coronal sections from rat hippocampus (**A**) rat nucleus tractus solitarius (**B**), or rat hippocampus (**C**) were incubated with either anti-5HT1AR (**A**), anti-AT1R (**B**), anti-OPRM (**C**) antibodies followed by incubation with Alexa-labeled secondary antibodies (green) as described in Methods. Propidium iodine (red) was used as a nuclear stain. White arrows–red staining associated with receptor staining (green); white arrowheads–red staining not associated with receptor staining.
(EPS)

S5 Fig Endogenous receptor recognition by anti-GPCR antibodies. SK-N-SH cells were treated with different concentrations (0–10 μM) of either quinpirole (**A**), DPAT (**B**), cholecystokinin 8 (CCK8) (**C**), ghrelin (**D**), DAMGO (**E**), bradykinin (**F**) or substance P (**G**) and changes in receptor recognition were measured by ELISA using either anti-DRD2 (**A**), anti-HTR1A (**B**), anti-CCK1R (**C**), anti-GHSR (**D**), anti-OPRM (**E**), anti-BKR2 (**F**) or anti-NKR1 (**G**) antibodies as described in Methods. Values obtained with vehicle treatment were taken as 100%. Data represent Mean ± SE, n = 3.
(EPS)

S1 Table Receptor recognition by anti-GPCR antibodies. HEK293 cells alone (HEK293) or expressing individual receptors (HEK293+ receptor) were subjected to ELISA using anti-receptor antibodies as described in Methods. Data represent Mean ± SE, n = 3–6.
(DOCX)
