# Correction: Generation of G protein-coupled receptor antibodies differentially sensitive to conformational states

**DOI:** 10.1371/journal.pone.0193322

**Published:** 2018-02-15

**Authors:** Andrea S. Heimann, Achla Gupta, Ivone Gomes, Rayees Rahman, Avner Schlessinger, Emer S. Ferro, Ellen M. Unterwald, Lakshmi A. Devi

The fourth author’s name is incorrect. The correct name is: Rayees Rahman. The correct citation is: Heimann AS, Gupta A, Gomes I, Rahman R, Schlessinger A, Ferro ES, et al. (2017) Generation of G protein-coupled receptor antibodies differentially sensitive to conformational states. PLoS ONE 12(11): e0187306. https://doi.org/10.1371/journal.pone.0187306
