# Supplementary material for: Generation of G protein-coupled receptor antibodies differentially sensitive to conformational states
Source: PLoS One. 2017 Nov 1;12(11):e0187306. doi: 10.1371/journal.pone.0187306 (PMC5665533; doi:10.1371/journal.pone.0187306)
Supplement: S1 Table — HEK293 cells alone (HEK293) or expressing individual receptors (HEK293+ receptor) were subjected to ELISA using anti-receptor antibodies as described in Methods. Data represent Mean ± SE, n = 3–6. (DOCX) [file pone.0187306.s006.docx]

| **Receptor Name** | **Antigen** | HEK293 | HEK293+ receptor |
| --- | --- | --- | --- |
| **D2 dopamine** | LSWYDDDLER | 0.0880±0.0074 | 0.1143±0.0086 |
| **Mu opioid** | SDPLAPASWSPAPGSWL | 0.0783±0.0030 | 0.1073±0.0046 |
| **Delta opioid** | LVPSARAELQSS | 0.0778±0.0066 | 0.1097±0.0014 |
| **MC4 melanocortin** | NSTHHHGMYTSLHLWN | 0.0774±0.0037 | 0.1007±0.0021 |
| **B1 bradykinin** | QAPANITSCE | 0.0774±0.0033 | 0.1082±0.0029 |
| **Beta2 adrenergic** | SRAPDHDVTQE | 0.0800±0.0046 | 0.1273±0.0039 |
| **AT1 angiotensin** | lnsstedgik | 0.0799±0.0060 | 0.1990±0.0047 |
| **GPCR55** | PTLSQLDSN | 0.0683±0.0065 | 0.1794±0.0047 |
| **Ghrelin (GHSR)** | DLDWDASPGN | 0.0853±0.0011 | 0.1789±0.0047 |
| **CB1 cannabinoid** | DIQYEDIKGDMA | 0.0726±0.0051 | 0.1803±0.0046 |
| **5HT_1A_ serotonin** | TTTSLEPFGTG | 0.0856±0.0034 | 0.1792±0.0044 |
| **Neurotensin** | MEATFLALSL | 0.0876±0.0073 | 0.1791±0.0044 |
| **Vasopressin V1b** | SEPSWTATPS | 0.0736±0.0061 | 0.1822±0.0046 |
| **Cholecystokinin 1** | VVDSLLMNGSNI | 0.0900±0.0036 | 0.1793±0.0044 |
| **Beta1 adrenergic** | PLPDGAATAARL | 0.07993±0.0056 | 0.2553±0.0039 |
| **B2 bradykinin** | NCPDTEWWSWLNA | 0.0697±0.0030 | 0.1795±0.0047 |
| **a2A adenosine** | GTEAPGGGTRATPYS | 0.0789±0.0041 | 0,1779±0.0043 |
| **M2 muscarinic** | NNGLAITSPY | 0.0966±0.0033 | 0.1768±0.0041 |
| **CB2 cannabinoid** | GLEFNPMKEYMI | 0.0590±0.0070 | 0.1776±0.0040 |
| **NK1 (substance P)** | VLPMDSDLFP | 0.0745±0.0034 | 0.1754±0.0038 |
| **Neuropeptide Y1** | TLFSRVENYSVHYNV | 0.0712±0.0023 | 0.1755±0.0037 |
| **AT2 angiotensin** | DNFSFAATSR | 0.0778±0.0067 | 0.1752±0.0040 |
| **Alpha1b adrenergic** | DLDTGHNTSAPAH | 0.0810±0.0040 | 0.1728±0.0034 |
| **Kappa opioid** | DQQLEPAHISPA | 0.0699±0.0035 | 0.1752±0.0031 |
| **D1 dopamine** | NTSTMDEAGLPAERDF | 0.0710±0.0023 | 0.1756±0.0032 |
| **Mu opioid** | SDPLAPASWSPA | 0.0756±0.0012 | 0.1730±0.0032 |
| **M1 muscarinic** | LAPGKGPWQV | 0.0750±0.0037 | 0.1728±0.0037 |
| **CB2 cannabinoid** | CRELELTNGSN | 0.0812±0.0055 | 0.1732±0.0040 |
| **Endothelin (EDNRB)** | EVMTPPTKT | 0.0775±0.0011 | 0.1734±0.0040 |
| **NK1 (substance P)** | SNQFVQPTWQ | 0.0479±0.0010 | 0.1744±0.0042 |
| **Leukotriene B4R (BLTR1)** | TAATSSPGGM | 0.0765±0.0034 | 0.1745±0.0042 |
| **Leukotriene B4R2 (BLTR2)** | SVCYRPPGNE | 0.0843±0.0061 | 0.1762±0.0039 |
| **Prostaglandin D2** | NESYRCQTSTWV | 0.0716±0.0094 | 0.1743±0.0042 |
| **Prostaglandin E2** | NSFNDSRRVE | 0.0801±0.0061 | 0.1737±0.0046 |
| **Prostaglandin F2** | SINSSKQPAS | 0.0767±0.0022 | 0.1769±0.0047 |
| **Vasopressin V1a** | DVRNEELAKL | 0.0899±0.0037 | 0.1770±0.0045 |
| **Vasopressin V2** | STVSAVPG | 0.0643±0.0032 | 0.1758±0.0035 |
| **Neurotensin** | LDVNTDIYSKVLVT | 0.0722±0.0023 | 0.1766±0.0028 |
